# Reducing manual workload in CT and MRI annotation with the Segment Anything Model 2

**DOI:** 10.1186/s12880-025-02075-4

**Published:** 2026-01-08

**Authors:** Leo Misera, Sven Nebelung, Zunamys I. Carrero, Keno Bressem, Marta Ligero, Jens-Peter Kühn, Ralf-Thorsten Hoffmann, Daniel Truhn, Jakob Nikolas Kather

**Affiliations:** 1 Institute and Polyclinic for Diagnostic and Interventional Radiology, Faculty of Medicine and University Hospital Carl Gustav Carus Dresden, Technical University Dresden, Dresden, Germany; 2 Else Kroener Fresenius Center for Digital Health, Technical University Dresden, Dresden, Germany; 3 Department of Diagnostic and Interventional Radiology, University Hospital Aachen, Aachen, Germany; 4 Department of Diagnostic and Interventional Radiology, Klinikum Rechts der Isar, Technical University of Munich, School of Medicine and Health, TUM University Hospital, Munich, Germany; 5 Department of Cardiovascular Radiology and Nuclear Medicine, Technical University of Munich, School of Medicine and Health, German Heart Center, TUM University Hospital, Munich, Germany; 6 Department of Medicine I, Faculty of Medicine and University Hospital Carl Gustav Carus, Technical University Dresden, Dresden, Germany; 7 Medical Oncology, National Center for Tumor Diseases (NCT), University Hospital Heidelberg, Heidelberg, Germany; 8 Pathology & Data Analytics, Leeds Institute of Medical Research at St James’s, University of Leeds, Leeds, UK

**Keywords:** Segment anything model, Deep learning, Segmentation, CT, MRI, Image annotation, Radiology

## Abstract

**Background:**

Volumetric segmentation in CT and MRI is valuable for artificial intelligence workflows in radiology, yet creating the large, precisely annotated datasets required for training segmentation models remains laborious.

**Methods:**

Here, we tested in simulation whether the foundation model “Segment Anything Model 2” (SAM 2) can reduce expert annotation workload. In our workflow, annotators provide a single box at the object’s center, and SAM 2 automatically segments the object across slices; annotators then review and correct the masks as needed. Workload reduction was defined as the proportion of SAM 2’s predicted segmentation masks that were accepted without modification. Downstream segmentation models were then trained on the SAM 2-assisted masks and compared with reference models trained on ground truth masks.

**Results:**

For femoral bone segmentation in MRI and liver tumor segmentation in CT, 36,614 sagittal and 16,311 axial slices were annotated, with 30% and 53% of SAM 2-generated masks accepted as is, respectively, indicating workload reduction. Crucially, segmentation models trained on SAM 2-assisted masks performed comparably to reference models, with a median dice similarity coefficient of 98.5% compared with 98.7% for femoral bone segmentation, and 77.3% compared with 77.0% for liver tumor segmentation.

**Conclusion:**

Using SAM 2 could thus expedite 3D medical imaging dataset annotation and segmentation model development for both research and clinical applications.

## Introduction

Volumetric segmentation of anatomical structures or lesions from 3D imaging data, such as computed tomography (CT) and magnetic resonance imaging (MRI), is widely used in clinical practice and research, including volumetric assessment [1, 2], texture analysis [3, 4], and radiation planning [5, 6]. However, creating these segmentation masks relies on expert annotators, such as radiologists or radiation oncologists, who must manually delineate each object, a process that is both time-consuming and resource-intensive [7]. For instance, delineating organs at risk around a tumor for radiation planning can take several hours per patient [8, 9], potentially leading to treatment delays and worse clinical outcomes [10]. Deep learning-based automatic segmentation offers a promising solution to support physicians and researchers in these tasks [11]. However, training such models requires large datasets [12], with precise object delineations, which are often created through manual annotation. Thus, reducing annotation workload is crucial.

**Fig. 1:**
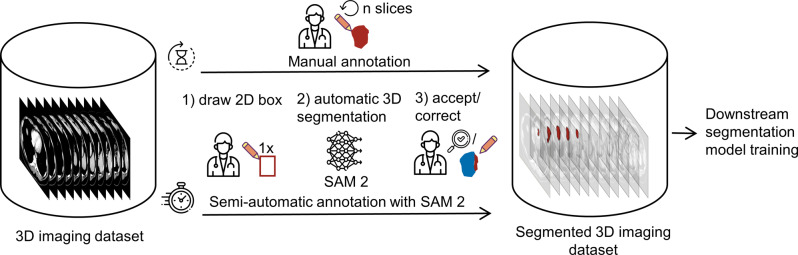
Annotation workflows, illustrated for liver tumor segmentation in CT. In the manual workflow, the user annotates each tumor slice-by-slice (upper arrow), which is inherently laborious. The SAM-2 assisted annotation workflow (lower arrow) requires only little user input during annotation. The segmentation outlines enable training downstream segmentation models, for example for research or clinical use. CT adapted from [17, 18]

One approach to reducing annotation effort is using promptable foundation models, such as Segment Anything Model (SAM 1) [13], which accepts sparse inputs [14, 15], such as dots or bounding boxes. These sparse annotations prompt the model to generate precise segmentation masks. Its successor, Segment Anything Model 2 (SAM 2) [16], extends this capability by propagating generated segmentation masks across frames in a video or slices in a cross-sectional image volume, thus potentially reducing manual workload in annotating 3D medical imaging data (Fig. 1).

Recent technical studies [19, 20] have shown that prompting SAM 2 with a single bounding box at the object’s center is an efficient method for generating segmentation masks across slices for various anatomical structures and lesion types in 3D CT and MRI. In a real-world SAM 2-assisted annotation workflow, an annotator would first select the center slice of an object (e.g., a lesion) and draw a single bounding box, from which SAM 2 automatically generates segmentation masks across slices in the 3D volume. Instead of manually segmenting each slice, the annotator would then only need to review and correct segmentation masks where SAM 2’s predictions were inaccurate (Fig. 1). However, it remains unclear to what extent the manual workload associated with annotating large training datasets for downstream segmentation model training can be reduced by using the SAM 2-assisted annotation workflow.

To address this, we investigated what proportion of SAM 2’s predicted segmentation masks can be accepted without modification while maintaining the performance of downstream segmentation models trained on these masks. To this end, we performed a SAM 2-assisted annotation workflow with varying levels of user interaction and trained segmentation models using the resulting masks. This evaluation was conducted across two representative use cases: femoral bone segmentation in MRI and liver tumor segmentation in CT. By analyzing the relationship between the proportion of accepted masks and downstream model performance, we evaluated SAM 2’s potential to reduce manual annotation workload. This study, in turn, serves as a proof of concept for integrating SAM 2 into 3D medical imaging annotation workflows. Our findings indicate that SAM 2 can substantially reduce manual workload in CT and MRI dataset annotation for segmentation model training, with promising implications for both clinical and research applications.

## Materials and methods

**Fig. 2:**
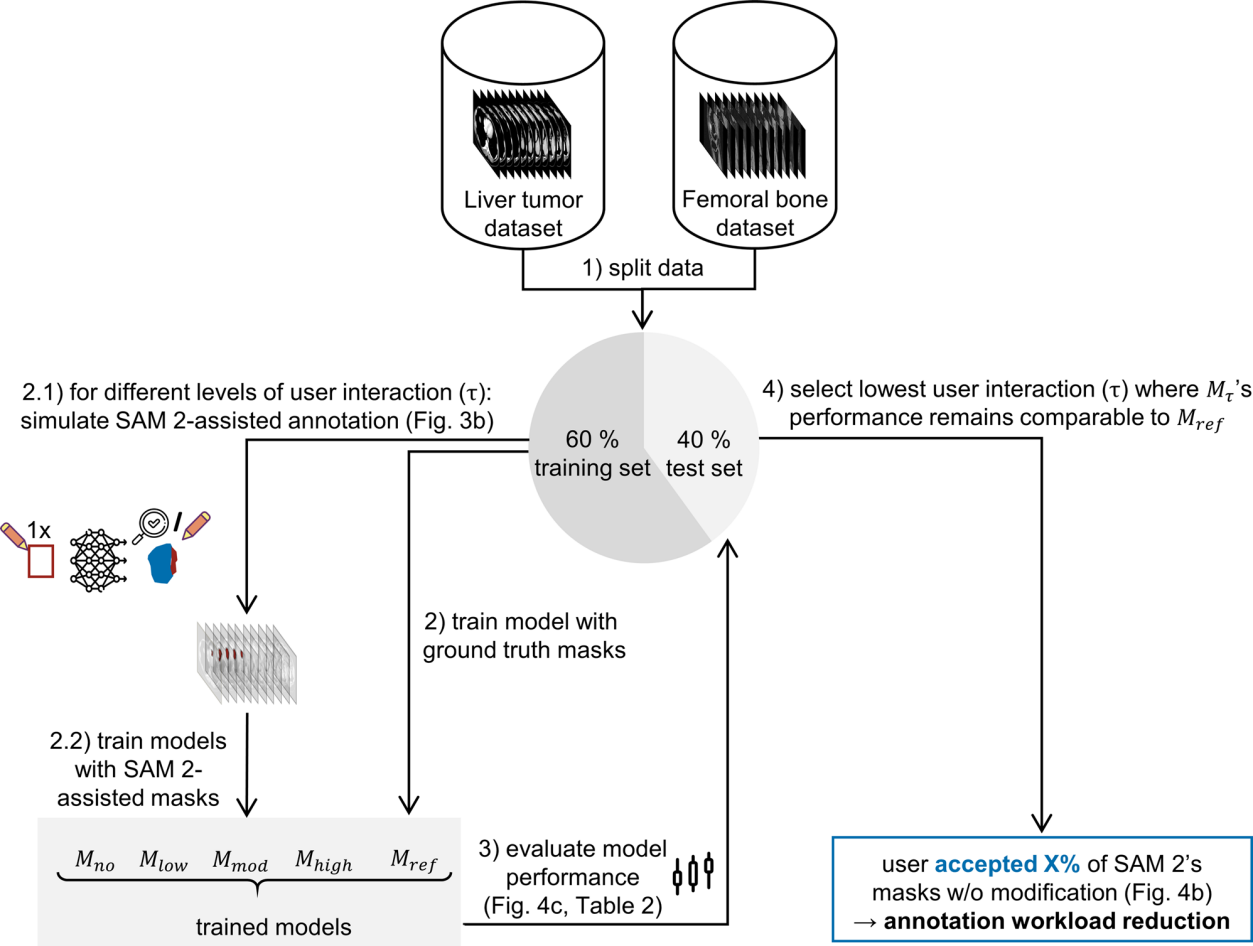
Study design measuring manual annotation workload reduction via the proportion of SAM 2-generated masks accepted as is without compromising downstream segmentation model performance. CT and MRI adapted from [17, 18, 21–24]

We evaluated whether SAM 2 can reduce annotation workload while maintaining downstream segmentation model performance. The study comprised four main steps: data preprocessing, simulating SAM 2-assisted annotation under varying degrees of user interaction, training downstream segmentation models on the SAM 2-assisted segmentation masks, and evaluating workload reduction and model performance compared to models trained on ground truth segmentation masks (Fig. 2).

## Datasets

For femoral bone segmentation, the OAI-ZIB dataset [21–24] was used. This dataset comprised MRI scans from 507 patients with ground truth segmentation masks. Note that only 505 patients were included while two MRI scans, (IDs 9539084 and 9445105), were excluded due to missing or implausible ground truth segmentation masks. To remove small, disconnected regions erroneously marked as femoral bone beyond the actual bone in a small number of ground-truth masks, only the largest connected volume corresponding to the femoral bone was retained; smaller connected regions were reclassified as background.

For liver tumor segmentation, we combined the liver tumor segmentation dataset [25] (LiTS), the colorectal liver metastases dataset [17] (CLM) and the WAW-TACE dataset [26] (WAW) to increase the sample size. From LiTS, we excluded any CT scans without a segmented tumor, reducing the original 131 scans to 118. From CLM, which initially had 197 CT scans, we excluded two scans (IDs 1187 and 1034) because of misaligned segmentation masks. The WAW dataset contained 233 CT scans acquired in multiple phases per patient, but segmentation was available for only one phase. We included only the phase with a corresponding segmentation mask, while native (non-contrast-enhanced) scans were excluded (IDs 13, 458, 501).

## Simulation of SAM 2-assisted annotation

Prior to using SAM 2, we preprocessed the scans. For the CT scans, we extracted axial slices, clipped intensities to −55 and 155 Hounsfield Units, following [27], and scaled the resulting intensities between 0 and 255. For the MRI scans, we extracted the sagittal slices, clipped intensities at the 1st and 99th percentiles to remove extreme values, and scaled the resulting intensities to values between 0 and 255.

Before simulating the annotation workflow, we first compared two prompt modes, a center dot and a bounding box, to determine which provided more accurate segmentations as the basis for subsequent experiments. The center dot or bounding box was placed on the object’s center slice, which was identified by computing the center of mass of the object’s 3D ground truth segmentation mask. To mimic realistic user imprecision, the dot and box prompts were slightly varied. For the dot prompt, the centroid of the 2D ground truth segmentation mask was computed and then shifted by up to ± 2 mm, but not more than 10% of the object’s maximum width or height, ensuring the dot remained within the object. For the box prompt, a bounding box was derived from the 2D ground truth segmentation mask and then slightly varied in size and position. The box width and height were independently scaled by up to ± 2 mm, but by no more than 5% of their width or height, and additionally shifted by up to ± 1 mm, but not more than 5% of their width or height. SAM 2 then generated a segmentation mask for the object’s center slice based on either prompt. Segmentation mask quality was measured using the dice similarity coefficient (DSC), and statistical differences between the two prompt types were tested with two-tailed Wilcoxon signed-rank tests. In practice, this setup reflects how a user might roughly mark an object’s center with a dot or draw a bounding box, with small placement errors.

**Fig. 3:**
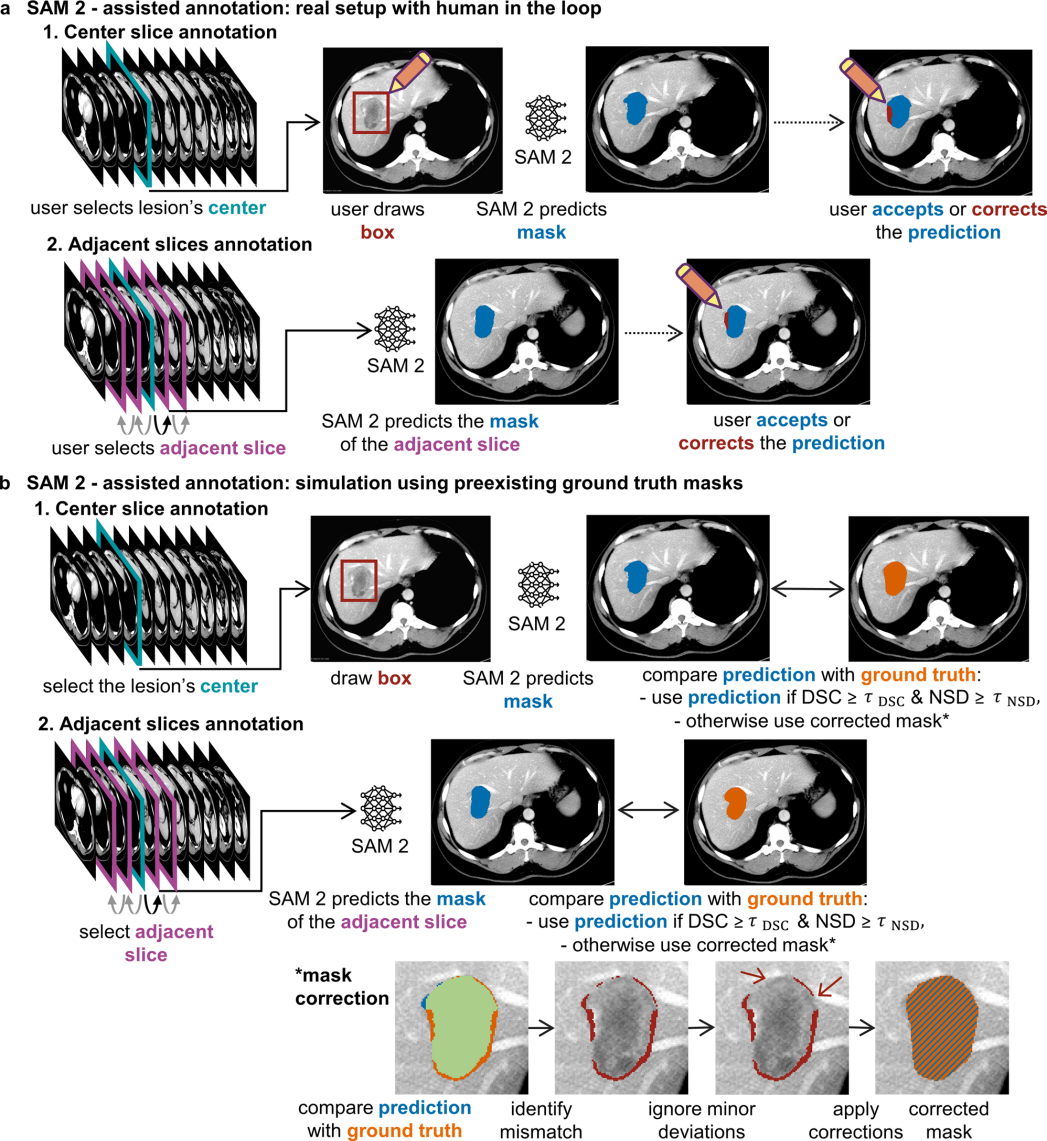
(**a**) real world SAM 2-assisted annotation workflow illustrated for liver tumor segmentation. (**b**) simulated workflow using ground truth masks, with dice similarity coefficient (DSC) and normalized surface dice at 1 mm tolerance (NSD) measuring segmentation mask quality and threshold τ controlling user interaction and workload. CT adapted from [17, 18]

For subsequent experiments, only the box prompt was used. The real annotation workflow was simulated as follows (Fig. 3a, b). First, the center slice of each target object (e.g., the femoral bone in an MRI or an individual liver tumor in a CT) was selected. SAM 2 was then prompted with a bounding box, based on which SAM 2 then segmented the object slice-by-slice from the object’s center to its boundaries. In practice, a human annotator would review each of SAM 2’s predicted 2D segmentation masks and decide whether to accept or correct them. In our simulation, this decision was approximated using two quantitative criteria: the DSC to measure overlap and the normalized surface dice with 1 mm tolerance (NSD) to measure boundary agreement against the respective 2D ground truth mask. SAM 2’s predicted segmentation mask was accepted only if both the DSC and NSD exceeded their respective threshold; otherwise, it was corrected. To avoid the unrealistic assumption of perfect corrections, a human-like strategy was implemented: areas where the predicted and ground truth segmentation masks differed were identified with an element-wise XOR operation and grouped into connected regions. A region was corrected only if at least one voxel lay more than 1 mm away from the ground truth boundary, thereby mimicking a user who corrects major errors while tolerating minor inaccuracies (Fig. 3b, bottom). The resulting corrected segmentation mask was used both as the final segmentation mask for training the respective downstream model and to re-prompt SAM 2 for correction. For volumes containing multiple objects, such as several liver tumors, the workflow was applied separately to each object. Notably, SAM 2’s state was reset before each new bounding box prompt or correction prompt.

To explore different degrees of user involvement, four simulation scenarios were defined. In all scenarios, the user provided a bounding box in the object’s center and indicated the object’s longitudinal extent. The first scenario considered no further manual segmentation mask edits, i.e. the user accepted all of SAM 2’s predicted segmentation masks within the object’s longitudinal extent. In the other three scenarios, the annotator corrected SAM 2’s predicted segmentation masks if necessary, reflecting low, moderate, and high degrees of user interaction.

To define thresholds for the four simulation scenarios, we measured how often corrections were required under different acceptance thresholds of DSC or NSD. For the femoral bone dataset, DSC thresholds ranging from 0.90 to 0.995 were evaluated while NSD was fixed at zero, and NSD thresholds ranging from 0.70 to 0.99 were evaluated while DSC was fixed at zero. For the liver tumor dataset, DSC thresholds ranged from 0.30 to 0.95, and NSD thresholds from 0.10 to 0.90, each again evaluated with the other threshold fixed at zero. For each threshold setting, the proportion of instance slices that required correction was recorded. Scenario-specific thresholds ${\tau _{no}}$, ${\tau _{low}}$, ${\tau _{mod}}$, and ${\tau _{high}}$ were then derived by linear interpolation so that approximately 0%, 20%, 40%, and 60% of instance slices required correction, corresponding to no edits, low, moderate, and high degree of user interaction. Suitable threshold values were obtained for DSC and NSD separately, but both were applied jointly in the downstream simulations to determine segmentation mask quality.

All simulations were performed on a local computer with a NVIDIA RTX 6000 GPU using python 3.11.12. We used SAM 2 version 1.0 with the sam2_hiera_tiny model throughout the simulation of SAM 2-assisted annotation.

## Downstream segmentation model training and performance evaluation

The nn-UNet framework [28] (version 2.2.1) was used for downstream segmentation model training due to its proven effectiveness on numerous medical segmentation tasks [11]. We split each dataset into a training set (60%) and a test set (40%). Each downstream model employed the 3D_fullres architecture and was trained for 150 epochs using five-fold cross-validation with a batch size of 8.

For liver tumor segmentation, we first cropped each CT volume to a rectangular cuboid enclosing the liver based on liver masks automatically obtained via TotalSegmentator [29] (version 2.4.0) to remove irrelevant areas of each CT scan before training the nn-UNet, and clipped intensities to −55 to 155 Hounsfield units [27]. For femoral bone segmentation, we applied z‑score normalization to the MRI scans and did not crop the volumes.

We evaluated model performance on the test sets using the preexisting ground truth segmentation masks. For each dataset, a reference model (${M_{ref}}$) was trained using the ground truth segmentation masks from the training set. Additional models (${M_{no}}$, ${M_{low}}$, ${M_{mod}}$ and ${M_{high}}$) were trained using segmentation masks derived from the SAM 2-assisted annotation simulation at different thresholds τ. Model performance was quantified by the DSC and NSD with a 1 mm tolerance [11, 30]. Here, both metrics were computed based on the 3D segmentation masks. To determine significant performance differences between segmentation models trained with SAM 2‑generated segmentation masks ${M_\tau }$ and the ground truth model ${M_{ref}}$, we conducted two-tailed Wilcoxon signed‑rank tests with Holm correction applied separately for each dataset.

## Results

### Datasets

To evaluate the extent to which SAM 2 reduces manual annotation workload while maintaining performance of downstream segmentation models, we conducted experiments on two datasets: the femoral bone dataset (OAI-ZIB [21–24]) and the liver tumor dataset (LiTS [25]; CLM [17, 18], WAW [26]) (Table 1). The femoral bone dataset comprised 505 3D MRI scans, one per patient, with preexisting annotations delineating the femoral bone. Of these, 303 scans (60%) were selected as the training set to simulate the annotation workflow and train downstream segmentation models, containing 36,614 sagittal slices with femoral bone. The remaining 202 scans (40%) were used to test downstream segmentation model performance. The liver tumor dataset contained 543 3D CT scans, one per patient. Of these, 326 scans (60%), including 997 tumors, were designated for training. This subset contained 12,085 axial slices that showed at least one tumor. Because multiple tumors can appear on the same slice, the total number of individual tumor instance slices requiring annotation was 16,311. The remaining 217 scans (40%), comprising 795 tumors, were selected to test downstream model performance.

**Table 1:** Main dataset characteristics. The statistics refer to the data used in the experiments without the excluded samples

Dataset	Osteoarthritis Initiative (OAI-ZIB) [21–24]	Liver Tumor Segmentation train set (LiTS) [25]	Colorectal-Liver-Metastases (CLM) [17, 18]	WAW-TACE (WAW) [26]
Number of patients	505	118	195	230
Imaging modality	MRI (DESS sequence)	Contrast-enhanced CT	Contrast-enhanced CT	Contrast-enhanced CT
Segmentation masks	Femoral bone	Liver tumor	Liver tumor	Liver tumor
Image resolution in-plane (px)	384 ×384	512 ×512	512 ×512	512 ×512 or 768 × 768
Orientation	Sagittal	Axial	Axial	Axial
Slice thickness: mean [min, max] (mm)	0.7 [0.7, 0.7]	1.57 [0.7, 5]	2.18 [0.8, 7.5]	1.76 [0.63, 5]

Notably, the LiTS, CLM and WAW dataset were combined, comprising the liver tumor dataset

### Simulation of SAM 2-assisted annotation

In a preliminary comparison of prompt modes applied to the center slice of each object, bounding boxes substantially outperformed central dots. For these center slices, the median and interquartile range (IQR) of the DSC was 94.5% (IQR: 93.2% − 95.5%) compared with 42.3% (IQR: 24.6% − 70.9%) for the center slices of the femoral bone dataset (*n* = 303), and 85.7% (IQR: 79.2% − 90.2%) compared with 3.9% (IQR: 0.7% − 70.4%) for the center slices of the liver tumor dataset (*n* = 997), with both differences significant (both *p* < 0.001). All subsequent experiments therefore used bounding boxes for prompting the center slice of each object.

**Fig. 4:**
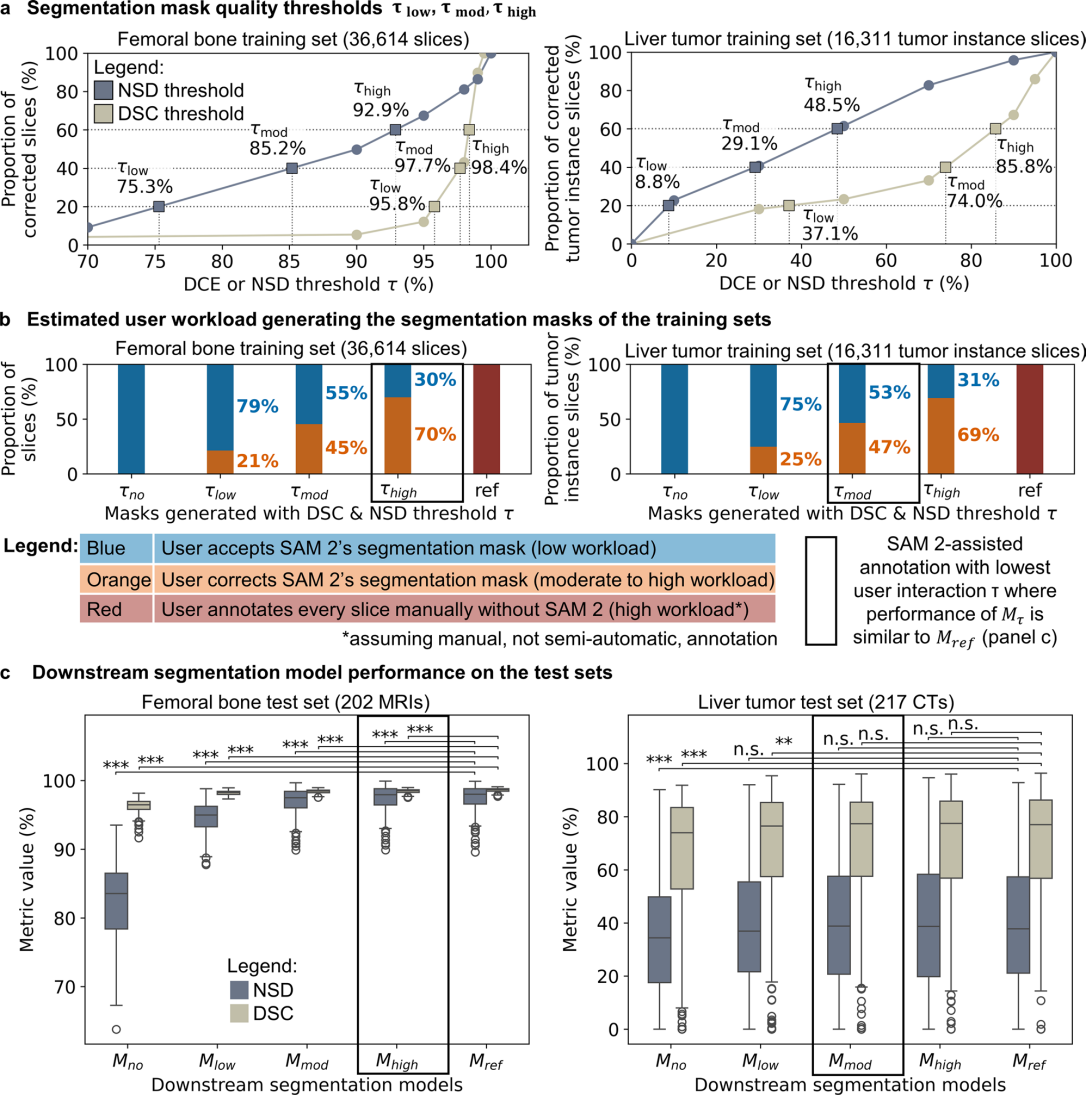
(**a**) segmentation mask quality dice similarity coefficient (DSC) and normalized surface dice at tolerance 1 mm (NSD) thresholds ${\tau _{low}}$, ${\tau _{mod}}$ and ${\tau _{high}}$, obtained through interpolation, corresponding to low, moderate, high degree of user interaction. (**b**) estimated manual user workload for annotating all slices in the training set. (**c**) test set performance of the downstream segmentation models trained with SAM 2-assisted segmentation masks (${M_{no}}$, ${M_{low}}$, ${M_{mod}}$ and ${M_{high}}$) and ground truth segmentation masks (${M_{ref}}$). The box plots’ whiskers extend to 1.5× of the interquartile range. P-values, adjusted using Holm method for each dataset, compare model performance using Wilcoxon signed-rank test: *p* ≥ 0.5 (n.S.), *p* < 0.05 (*), *p* < 0.01 (**), *p* < 0.001 (***)

For each dataset, thresholds for the four scenarios (no edits, low, moderate, and high degree of user interaction) were calibrated by simulating the annotation workflow across a range of DSC and NSD thresholds, separately, measuring the proportion of corrected masks. In the femoral bone dataset, ${\tau _{low}}$ was defined as 95.8% for DSC and 75.3% for NSD, ${\tau _{mod}}$ as 97.7% and 85.2%, and ${\tau _{high}}$ as 98.4% and 92.9%, respectively (Fig. 4a, left). In the liver tumor dataset, ${\tau _{low}}$ was defined as 37.1% for DSC and 8.8% for NSD, ${\tau _{mod}}$ as 74.0% and 29.1%, and ${\tau _{high}}$ as 85.8% and 48.5%, respectively (Fig. 4a, right). For the no edits scenario, ${\tau _{no}}$, both DSC and NSD thresholds were set to zero for each dataset. In the downstream simulations, the respective DSC and NSD thresholds were applied jointly, such that a segmentation mask predicted by SAM 2 was accepted only if it exceeded both thresholds when compared to the ground truth.

The annotation simulation was then performed for the four scenarios defined by the above calculated thresholds ${\tau _{no}}$, ${\tau _{low}}$, ${\tau _{mod}}$, and ${\tau _{high}}$, corresponding to no edits, low, moderate, and high degree of user interaction, respectively (Fig. 2).

For femoral bone segmentation, to create the training dataset comprising 303 MRI scans, 36,614 sagittal MRI slices required annotation. The user accepted 100%, 79%, 55%, and 30% of SAM 2’s masks without modification at ${\tau _{no}}$, ${\tau _{low}}$, ${\tau _{mod}}$, and ${\tau _{high}}$, respectively, while corrections were required for the remaining 0%, 21%, 45%, and 70% (Fig. 4b, left).

For liver tumor segmentation, to create the training dataset comprising 326 CT scans, 16,311 tumor instance slices required annotation. The user accepted 100%, 75%, 53%, and 31% of SAM 2’s masks without modification at ${\tau _{no}}$, ${\tau _{low}}$, ${\tau _{mod}}$, and ${\tau _{high}}$, respectively, while corrections were required for the remaining 0%, 25%, 47%, and 69% (Fig. 4b, right).

### Downstream model training & assessment of workload reduction

Next, we determined the extent to which manual annotation workload (i.e., user interaction) can be reduced while maintaining downstream segmentation model performance. To this end, using segmentation masks generated at each degree of user interaction (${\tau _{no}}$, ${\tau _{low}}$, ${\tau _{mod}}$, ${\tau _{high}}$), we trained downstream segmentation models (${M_{no}}$, ${M_{low}}$, ${M_{mod}}$ and ${M_{high}}$) and compared their performance to that of the model trained on preexisting ground truth segmentation masks (${M_{ref}}$). Manual annotation workload reduction was quantified by identifying the lowest τ value at which the performance remained comparable to ${M_{ref}}$ on the test set (Fig. 2).

For femoral bone segmentation, only the downstream model trained with SAM 2-assisted segmentation masks at high annotator interaction ($${M_{high}}$$) achieved performance comparable to $${M_{ref}} $$ on the test set. $${M_{high}}$$ achieved a median DSC of 98.5% and median NSD of 97.9%, closely matching $${M_{ref}}$$’s DSC of 98.7% and NSD of 98.0% (Fig. 4c, left; Table 2, left).

**Table 2:** Downstream segmentation model performance on each test set, reported as the median and interquartile range (IQR) for dice similarity coefficient (DSC) and normalized surface dice at tolerance 1 mm (NSD)

	Femoral bone dataset (*n* = 202)					Liver tumor dataset (*n* = 217)				
Model	${M_{no}}$	${M_{low}}$	${M_{mod}}$	${M_{high}}$	${M_{ref}}$	${M_{no}}$	${M_{low}}$	${M_{mod}}$	${M_{high}}$	${M_{ref}}$
DSC	96.5% IQR: 95.8% − 97.0%	98.3% IQR: 98.0% − 98.4%	98.5% IQR: 98.2% − 98.6%	98.5% IQR: 98.3% − 98.6%	98.7% IQR: 98.4% − 98.8%	73.9% IQR: 52.8% − 83.5%	76.5% IQR: 57.5% − 85.3%	77.3% IQR: 57.6% − 85.5%	77.5% IQR: 56.9% − 85.9%	77.0% IQR: 56.8% − 86.3%
p	< 0.001	< 0.001	< 0.001	< 0.001	n/a	< 0.001	< 0.001	0.17	0.62	n/a
adj. p	< 0.001	< 0.001	< 0.001	< 0.001	n/a	< 0.001	0.001	0.69	1	n/a
NSD	83.5% IQR: 78.4% − 86.5%	95.0% IQR: 93.3% − 96.3%	97.5% IQR: 96.0% − 98.4%	97.9% IQR: 96.5% − 98.8%	98.0% IQR: 96.6% − 98.8%	34.4% IQR: 17.5% − 49.8%	36.9% IQR: 21.6% − 55.4%	38.9% IQR: 20.7% − 57.6%	38.7% IQR: 19.8% − 58.3%	37.8% IQR: 21.1% − 57.3%
p	< 0.001	< 0.001	< 0.001	< 0.001	n/a	< 0.001	0.04	0.97	0.43	n/a
adj. p	< 0.001	< 0.001	< 0.001	< 0.001	n/a	< 0.001	0.2	1	1	n/a

P-values were derived from pairwise comparison of DSC and NSD of model ${M_{no}}$, ${M_{low}}$, ${M_{mod}}$ and ${M_{high}}$ with ${M_{ref}}$ using Wilcoxon signed-rank test. Adjusted p-values were calculated through Holm correction for each dataset, separately

For liver tumor segmentation, the downstream models trained with SAM 2-assisted segmentation masks $${M_{mod}}$$ and $${M_{high}}$$ performed comparably to $${M_{ref}} $$on the test set. Of note, $${M_{mod}} $$, which required the least user interaction, achieved a median DSC of 77.3% and a median NSD of 38.9%, closely matching $${M_{ref}}$$’s 77.0% DSC and 37.8% NSD (Fig. 4c, right; Table 2, right).

### Analysis of SAM 2’s segmentation mask quality

**Fig. 5:**
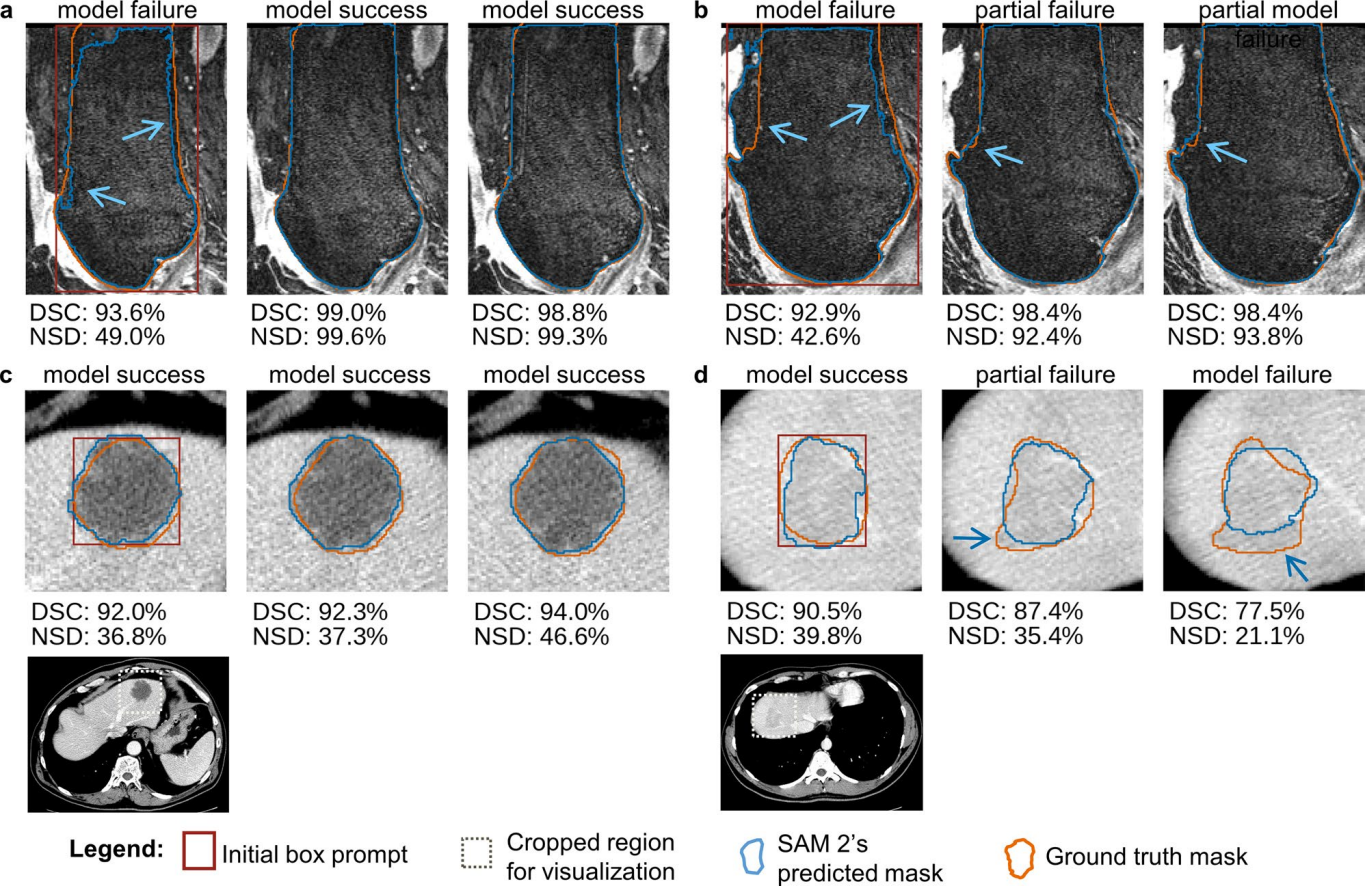
Visualization of adjacent slices and their segmentations, with segmentation mask quality measured by dice similarity coefficient (DSC) and normalized surface dice at tolerance 1 mm (NSD). The left image within each panel shows the center slice that was prompted with a bounding box. The middle and right image of each panel show the two subsequent slices and the respective predicted and ground truth mask. CTs and MRIs adapted from [17, 18, 21–24]

To further examine the quality of SAM 2-generated segmentations and identify cases requiring manual corrections, we visualized representative examples from τ settings that achieved the largest workload reduction maintaining downstream model performance, specifically ${\tau _{high}}$ for femoral bone segmentation and ${\tau _{mod}}$ for liver tumor segmentation (Fig. 5).

In cases where the foreground and background had a distinct boundary, SAM 2 consistently produced highly accurate segmentation masks requiring minimal or no correction at all (Fig. 5a, center and right; Fig. 5c). However, in low-contrast regions, predicted segmentation masks were often partially inaccurate, leading to increased manual correction efforts (Fig. 5a, left; Fig. 5b; Fig. 5d, center, right). These findings align with previous studies [19, 20, 31], which reported similar challenges in images with poor contrast. Nevertheless, even when the SAM 2-generated segmentation masks were partially inaccurate, a substantial portion of these segmentation masks were sufficiently accurate, likely reducing the effort needed for manual corrections compared to fully manual segmentation.

In summary, the annotator accepted 30% of SAM 2’s predicted segmentation masks without modification for femoral bone segmentation and 53% for liver tumor segmentation while preserving downstream segmentation model performance (Fig. 4b and c). These findings suggest that SAM 2-assisted annotation can substantially reduce manual annotation workload for both MRI and CT.

## Discussion

In this study, we investigated the potential of SAM 2, a general-purpose promptable segmentation model, to reduce manual annotation workload in 3D medical imaging data for two representative tasks, while maintaining the performance of downstream segmentation models. Our experiments on femoral bone segmentation in MRI and liver tumor segmentation in CT demonstrated that SAM 2 indeed substantially reduced manual annotation workload while maintaining downstream segmentation model performance. Notably, only inaccurate regions of the predicted segmentation masks required manual correction, suggesting that substantial reductions in manual workload are feasible.

One possible explanation for the comparable performance of downstream segmentation models trained with SAM 2-assisted segmentation masks is that differences between SAM 2-generated segmentation masks and ground truth segmentation masks may often fall within the range of typical inter-rater variability (Fig. 5a, center, right; Fig. 5c; Fig. 5d, left). Consistent with this, on the liver tumor dataset, ${M_{mod}}$, trained with SAM 2-assisted labels accepted above ${\tau _{mod}}$ (DSC threshold 74.0%, NSD threshold 29.1%), achieved performance comparable to ${M_{ref}}$. Prior work has reported a median inter-rater DSC of about 0.70 for liver tumor segmentation [25], closely matching threshold ${\tau _{mod}}$. In contrast, when labels with lower quality were included (${\tau _{no}}$, ${\tau _{low}}$), model performance declined. For the femoral bone dataset, no inter-rater variability has been reported. However, previous MRI studies on bone segmentation have reported a mean inter-rater DSC of 97.0% for manual femoral bone segmentation [32] and 98.8% for semi-automatic patella bone segmentation [33]. Consistent with this lower inter-rater variability, only ${M_{high}}$, trained with SAM 2-assisted labels of near ground truth quality (DSC threshold 98.4%, NSD threshold 92.9%), closely matched the performance of ${M_{ref}}$. Together, these findings suggest that SAM 2-assisted segmentation masks are suitable for downstream model training, provided that their quality remains within typical inter-rater variability.

Although the annotation process in our experiments was simulated, realistic user behavior was approximated. Bounding box prompts were deliberately varied in position and size to mimic user imprecision. In addition, when SAM 2’s predicted segmentation masks required correction, only major inaccuracies were edited while minor errors in SAM 2’s segmentation masks were left untouched, mimicking how users refine masks rather than redrawing them entirely (Fig. 3b, bottom). Although simulated, the workflow captures key aspects of practical annotation.

Overall, these findings indicate that SAM 2 could reduce manual annotation workload for other types of objects in 3D medical imaging data as well. As a result, more 3D images could be annotated in the same timeframe, thereby reducing the costs associated with training downstream segmentation models for research and medical device development. For example, accurate segmentations from these models could be applied to volumetric assessment [1, 2], texture analysis [3, 4], or radiation planning [5, 6]. SAM 2 also has a readily available plug-in for the widely used annotation tool 3D Slicer [34, 35], which facilitates easy integration into existing workflows.

This study has several limitations. First, we specifically focused on the default version of SAM 2 and did not include other available promptable segmentation models (e.g. [36–41]). This approach was chosen to maintain a consistent experimental design, as some alternatives to SAM 2 require other prompt modes or annotation workflows for optimum performance. Furthermore, foundation models trained on large datasets like SAM 2 can be applied zero-shot to other domains such as medical images [15, 19, 42, 43]. Importantly, to our knowledge, at the time of writing this manuscript, SAM 2 is openly available for both research and commercial use [44]. This stands in contrast to other models (e.g. [36–41]), which to our knowledge may remain restricted to research use only due to licenses of the training data. This made SAM 2 especially suitable for both academic and translational applications. Second, the annotation process in our experiments was simulated rather than performed by human annotators. While our design approximated realistic behavior, real annotators may place prompts differently, choose off-center slices for prompting, or rely on subjective rather than quantitative criteria for segmentation mask correction. Future user studies may therefore validate our findings under practical conditions, including quantifying the time savings using SAM 2 for both experienced and novice annotators and identifying cases where it is most effective. For instance, while large, well-defined structures may benefit from using SAM 2, very small or subtle targets could be faster to annotate from scratch. Third, we evaluated only two exemplary use cases, leaving open questions regarding SAM 2’s performance on other targets or imaging modalities. For example, certain anatomical structures with hollow or non-convex geometry like the myocardium may not be well suited for bounding box prompting and may require prompting strategies. Finally, we did not investigate whether SAM 2 introduces unintended biases, e.g. oversegmenting or undersegmenting certain structures, or whether performance differs between normal and pathological cases or across image quality. Any such biases could propagate into downstream models. To mitigate this risk, human annotators should always review SAM 2’s predicted segmentation masks, ensuring that systematic errors are identified and corrected. With appropriate human oversight, SAM 2 may therefore serve as a practical tool to reduce workload in real-world 3D medical imaging annotation workflows.

## Conclusion

In conclusion, our results suggest that SAM 2-assisted annotation can substantially reduce manual workload associated with annotating large 3D imaging datasets for downstream segmentation model training, while maintaining downstream model performance. Thus, integrating SAM 2 into existing annotation tools (e.g., via the openly available plug-in for 3DSlicer [34, 35]) holds promising potential for reducing manual workload in human segmentation workflows, ultimately saving costs and fostering further research and medical device development for clinical routine.

## Data Availability

The code and datasets used in the experiments can be accessed at the following sources: the SAM 2-assisted annotation simulation (https://github.com/KatherLab/3DAnnotationSimSAM2), the nn-UNet framework [28] for training downstream segmentation models (https://github.com/MIC-DKFZ/nnUNet), TotalSegmentator [29] for cropping the liver ROIs from CT scans (https://github.com/wasserth/TotalSegmentator), OAI-ZIB [21–24] dataset (https://huggingface.co/datasets/YongchengYAO/OAIZIB-CM), the LiTS [11, 25] dataset (http://medicaldecathlon.com/), the CLM dataset [17, 18] (https://www.cancerimagingarchive.net/collection/colorectal-liver-metastases/) and the WAW dataset [26] (https://zenodo.org/records/12741586).

## Abbreviations

CT: Computed Tomography
MRI: Magnetic Resonance Imaging
SAM 1: Segment Anything Model
SAM 2: Segment Anything Model 2
LiTS: Liver Tumor Segmentation Dataset
CLM: Colorectal Liver Metastases Dataset
WAW: WAW-TACE Dataset
DSC: Dice Similarity Coefficient
NSD: Normalized Surface Dice with 1 mm tolerance
${\tau _{no}}$, ${\tau _{low}}$, ${\tau _{mod}}$, and ${\tau _{high}}$: Threshold values used to define levels of user interaction (no, low, moderate, high) in SAM2-assisted annotation
${M_{ref}}$: Reference model, trained using the ground truth segmentation masks from the training set
${M_{no}}$, ${M_{low}}$, ${M_{mod}}$ and ${M_{high}}$: Model, trained using segmentation masks derived from the SAM 2-assisted annotation simulation at different thresholds τ

## References

[1] Aiudi D, Iacoangeli A, Dobran M, Polonara G, Chiapponi M, Mattioli A, et al. The prognostic role of volumetric MRI evaluation in the surgical treatment of glioblastoma. J Clin Med. 2024;13:849. 38337543 10.3390/jcm13030849 PMC10856584

[2] Koitka S, Kroll L, Malamutmann E, Oezcelik A, Nensa F. Fully automated body composition analysis in routine CT imaging using 3D semantic segmentation convolutional neural networks. Eur Radiol. 2021;31:1795–804. 32945971 10.1007/s00330-020-07147-3 PMC7979624

[3] Aerts HJWL, Velazquez ER, Leijenaar RTH, Parmar C, Grossmann P, Carvalho S, et al. Decoding tumour phenotype by noninvasive imaging using a quantitative radiomics approach. Nat Commun. 2014;5:4006. 24892406 10.1038/ncomms5006 PMC4059926

[4] Horvat N, Veeraraghavan H, Khan M, Blazic I, Zheng J, Capanu M, et al. MR imaging of rectal cancer: radiomics analysis to assess treatment response after neoadjuvant therapy. Radiology. 2018;287:833–43. 29514017 10.1148/radiol.2018172300 PMC5978457

[5] Harrison K, Pullen H, Welsh C, Oktay O, Alvarez-Valle J, Jena R. Machine learning for auto-segmentation in radiotherapy planning. Clin Oncol (R Coll Radiol). 2022;34:74–88. 34996682 10.1016/j.clon.2021.12.003

[6] Kashyap M, Wang X, Panjwani N, Hasan M, Zhang Q, Huang C, et al. Automated deep learning-based detection and segmentation of lung tumors at ct. Radiology. 2025;314:e233029. 39835976 10.1148/radiol.233029 PMC11783160

[7] Pullar-Strecker Z, Dost K, Frank E, Wicker J. Hitting the target: stopping active learning at the cost-based optimum. Mach Learn. 2024;113:1529–47.

[8] Harari PM, Song S, Tomé WA. Emphasizing conformal avoidance versus target definition for IMRT planning in head-and-neck cancer. Int J Radiat Oncol Biol Phys. 2010;77:950–58. 20378266 10.1016/j.ijrobp.2009.09.062 PMC2905233

[9] Hong TS, Tomé WA, Harari PM. Heterogeneity in head and neck IMRT target design and clinical practice. Radiother Oncol. 2012;103:92–98. 22405806 10.1016/j.radonc.2012.02.010 PMC3694728

[10] Chen Z, King W, Pearcey R, Kerba M, Mackillop WJ. The relationship between waiting time for radiotherapy and clinical outcomes: a systematic review of the literature. Radiother Oncol. 2008;87:3–16. 18160158 10.1016/j.radonc.2007.11.016

[11] Antonelli M, Reinke A, Bakas S, Farahani K, Kopp-Schneider A, Landman BA, et al. The medical segmentation decathlon. Nat Commun. 2022;13:4128. 35840566 10.1038/s41467-022-30695-9 PMC9287542

[12] Sun C, Shrivastava A, Singh S, Gupta A. Revisiting unreasonable effectiveness of data in deep learning era. 2017 IEEE International Conference on Computer Vision (ICCV). 2017. p. 843–52.

[13] Kirillov A, Mintun E, Ravi N, Mao H, Rolland C, Gustafson L, et al. Segment anything. arXiv [cs.CV]. 2023.

[14] Misera L, Müller-Franzes G, Truhn D, Kather JN. Weakly supervised deep learning in radiology. Radiology. 2024;312:e232085. 39041937 10.1148/radiol.232085

[15] Zhang Y, Zhao S, Gu H, Mazurowski MA. How to efficiently annotate images for best-performing deep learning-based segmentation models: an empirical study with weak and noisy annotations and segment anything model. J Imag Inf Med. 2025. 10.1007/s10278-025-01408-7. 10.1007/s10278-025-01408-7 PMC12572411 39843720

[16] Ravi N, Gabeur V, Hu Y-T, Hu R, Ryali C, Ma T, et al. Sam 2: segment anything in images and videos. arXiv [cs.CV]. 2024.

[17] Simpson AL, Peoples J, Creasy JM, Fichtinger G, Gangai N, Keshavamurthy KN, et al. Preoperative ct and survival data for patients undergoing resection of colorectal liver metastases. Sci Data. 2024;11:172. 10.1038/s41597-024-02981-2 PMC10847495 38321027

[18] Simpson AL, Peoples J, Creasy JM, Fichtinger G, Gangai N, Lasso A, et al. Preoperative ct and survival data for patients undergoing resection of colorectal liver metastases (colorectal-liver-metastases). 2023. 10.1038/s41597-024-02981-2 PMC10847495 38321027

[19] Dong H, Gu H, Chen Y, Yang J, Chen Y, Mazurowski MA. Segment anything model 2: an application to 2D and 3D medical images. arXiv [cs.CV]. 2024.10.1109/TBME.2026.365326741525616

[20] Ma J, Kim S, Li F, Baharoon M, Asakereh R, Lyu H, et al. Segment anything in medical images and videos: benchmark and deployment. arXiv [eess.IV]. 2024.

[21] Ambellan F, Tack A, Ehlke M, Zachow S. Automated segmentation of knee bone and cartilage combining statistical shape knowledge and convolutional neural networks: data from the osteoarthritis initiative. Med Image Anal. 2019;52:109–18. 30529224 10.1016/j.media.2018.11.009

[22] NDA. https://nda.nih.gov/oai. Accessed 17 Oct 2024.

[23] Yao Y, Chen W. Quantifying knee cartilage shape and lesion: From image to metrics. In: Lecture Notes in Computer Science. Cham: Springer Nature Switzerland; 2025. p. 162–72.

[24] Yao Y, Zhong J, Zhang L, Khan S, Chen W. CartiMorph: A framework for automated knee articular cartilage morphometrics. Med Image Anal. 2024;91:103035. 10.1016/j.media.2023.103035 37992496

[25] Bilic P, Christ P, Li HB, Vorontsov E, Ben-Cohen A, Kaissis G, et al. The liver tumor segmentation benchmark (LiTS). Med Image Anal. 2023;84:102680. 36481607 10.1016/j.media.2022.102680 PMC10631490

[26] Bartnik K, Bartczak T, Krzyziński M, Korzeniowski K, Lamparski K, Węgrzyn P, et al. WAW-TACE: a hepatocellular carcinoma multiphase ct dataset with segmentations, radiomics features, and clinical data. Radiol Artif Intell. 2024;6:e240296. 39441110 10.1148/ryai.240296 PMC11605144

[27] Wu L, Wang H, Chen Y, Zhang X, Zhang T, Shen N, et al. Beyond radiologist-level liver lesion detection on multi-phase contrast-enhanced CT images by deep learning. iScience. 2023;26:108183. 38026220 10.1016/j.isci.2023.108183 PMC10654534

[28] Isensee F, Jaeger PF, Kohl SAA, Petersen J, Maier-Hein KH. nnU-Net: a self-configuring method for deep learning-based biomedical image segmentation. Nat Methods. 2021;18:203–11. 33288961 10.1038/s41592-020-01008-z

[29] Wasserthal J, Breit H-C, Meyer MT, Pradella M, Hinck D, Sauter AW, et al. TotalSegmentator: robust segmentation of 104 anatomic structures in CT images. Radiol Artif Intell. 2023;5:e230024. 10.1148/ryai.230024 PMC10546353 37795137

[30] Maier-Hein L, Reinke A, Godau P, Tizabi MD, Buettner F, Christodoulou E, et al. Metrics reloaded: recommendations for image analysis validation. Nat Methods. 2024;21:195–212. 10.1038/s41592-023-02151-z PMC11182665 38347141

[31] Sengupta S, Chakrabarty S, Soni R. Is SAM 2 better than SAM in medical image segmentation? arXiv [eess.IV]. 2024.

[32] Xia Y, Fripp J, Chandra SS, Schwarz R, Engstrom C, Crozier S. Automated bone segmentation from large field of view 3D MR images of the hip joint. Phys Med Biol. 2013;58:7375–90. 10.1088/0031-9155/58/20/7375 24077264

[33] Heckelman LN, Soher BJ, Spritzer CE, Lewis BD, DeFrate LE. Design and validation of a semi-automatic bone segmentation algorithm from MRI to improve research efficiency. Sci Rep. 2022;12:7825. 10.1038/s41598-022-11785-6 PMC9098419 35551485

[34] Yildiz Z, Chen Y, Mazurowski MA. SAM & SAM 2 in 3D slicer: SegmentWithSAM extension for annotating medical images. arXiv [eess.IV]. 2024.

[35] Fedorov A, Beichel R, Kalpathy-Cramer J, Finet J, Fillion-Robin J-C, Pujol S, et al. 3D slicer as an image computing platform for the quantitative imaging network. Magn Reson Imag. 2012;30:1323–41. 10.1016/j.mri.2012.05.001 PMC3466397 22770690

[36] Archit A, Freckmann L, Pape C. MedicoSAM: towards foundation models for medical image segmentation. arXiv [eess.IV]. 2025.

[37] Zhu J, Hamdi A, Qi Y, Jin Y, Wu J. Medical SAM 2: segment medical images as video via segment anything Model 2. arXiv [cs.CV]. 2024.

[38] Ma J, He Y, Li F, Han L, You C, Wang B. Segment anything in medical images. Nat Commun. 2024;15:654. 10.1038/s41467-024-44824-z PMC10803759 38253604

[39] Bai Y, Yun B, Chen Z, Yu Q, Xia Y, Wang Y. RevSAM2: prompt SAM2 for medical image segmentation via reverse-propagation without fine-tuning. arXiv [cs.CV]. 2024.

[40] Lei W, Xu W, Li K, Zhang X, Zhang S. MedLSAM: localize and segment anything model for 3D CT images. Med Image Anal. 2025;99:103370. 10.1016/j.media.2024.103370 39447436

[41] Ma J, Yang Z, Kim S, Chen B, Baharoon M, Fallahpour A, et al. MedSAM2: segment anything in 3D medical images and videos. arXiv [eess.IV]. 2025.

[42] Aleem S, Wang F, Maniparambil M, Arazo E, Dietlmeier J, Curran K, et al. Test-time adaptation with SaLIP: a cascade of SAM and CLIP for zero-shot medical image segmentation. Proceedings of the IEEE/CVF Conference on Computer Vision and Pattern Recognition (CVPR) Workshops. 2024. p. 5184–93.

[43] Mattjie C, De Moura LV, Ravazio R, Kupssinskü L, Parraga O, Delucis MM, et al. Zero-shot performance of the segment anything model (SAM) in 2D medical imaging: a comprehensive evaluation and practical guidelines. 2023 IEEE 23rd International Conference on Bioinformatics and Bioengineering (BIBE). IEEE; 2023. p. 108–12.

[44] Introducing SAM 2: the next generation of meta segment anything Model for videos and images. https://ai.meta.com/blog/segment-anything-2/. Accessed 10 Apr 2025.

